# The combined effect of surface water and groundwater on environmental heterogeneity reveals the basis of beta diversity pattern in desert oasis communities

**DOI:** 10.1371/journal.pone.0279704

**Published:** 2022-12-27

**Authors:** Haobo Shi, Qingdong Shi, Hao Li, Xiaolong Zhou, Yue Dai, Yasenjiang Kahaer, Yanbo Wan, Lei Peng

**Affiliations:** 1 College of Ecology and Environment, Xinjiang University, Urumqi, 830046, China; 2 Key Laboratory of Oasis Ecology of Education Ministry, Xinjiang University, Urumqi, 830046, China; 3 Provost’s Office and Academic Affairs, Beijing Normal University at Zhuhai, Zhuhai, 519087, China; 4 College of Geography and Remote Sensing Sciences, Xinjiang University, Urumqi, 830046, China; Ningbo University, CHINA

## Abstract

Beta diversity indicates the species turnover with respect to a particular environmental gradient. It is crucial for understanding biodiversity maintenance mechanisms and for prescribing conservation measures. In this study, we aimed to reveal the drivers of beta diversity patterns in desert hinterland oasis communities by establishing three types of surface water disturbance and groundwater depth gradients. The results indicated that the dominant factor driving the beta diversity pattern within the same gradient shifted from soil organic matter to pH, as groundwater depth became shallower and surface water disturbance increased. Among the different gradients, surface water disturbance can have important effects on communities where original water resource conditions are extremely scarce. Under the premise that all habitats are disturbed by low surface water, differences in groundwater depth dominated the shifts in the community species composition. However, when groundwater depth in each habitat was shallow, surface water disturbance had little effect on the change in species composition. For the two components of beta diversity, the main drivers of species turnover pattern was the unique effects of surface water disturbance and soil environmental differences, and the main driver of species nestedness pattern was the common effect of multiple environmental pressures. The results of this study suggest that increasing the disturbance of surface water in dry areas with the help of river flooding will help in promoting vegetation restoration and alleviating the degradation of oases. They also confirm that surface water and groundwater mutually drive the establishment of desert oasis communities. Equal focus on both factors can contribute to the rational ecological recovery of dryland oases and prevent biodiversity loss.

## Introduction

The composition and distribution of species are the most fundamental characteristics of a community, and biodiversity research based on these two concepts is important within community ecology [1]. Species beta diversity is an indicator that represents differences in species composition between communities [2,3]. It indicates how species are separated by habitat and compares habitat diversity in different sections. For biodiversity conservation, the overall status of communities and ecosystems is often assessed based on species diversity characteristics, and then appropriate conservation strategies are developed [4–6]. The study of species diversity can reveal the relationship between species and their environment and enables us to elucidate the mechanisms of community assembly in heterogeneous habitat by understanding the role of different ecological processes in forming diversity patterns [7].

Current metrics on beta diversity can be conceptually divided into two distinct categories, proportional diversity, and differentiation diversity. Proportional diversity, often correlated with alpha and gamma diversity, relies on sampling units or spatio-temporal scales to compare differences in richness, such as multiplicative partitioning [8] and additive partitioning [9]. Differentiation diversity is used to compare the similarity of different sites under the prerequisite of considering the specific species composition of the community [10], such as the Jaccard and Sørensen indices [11,12]. With the advancements in research, the results of the differentiation index analysis is presumed to be influenced by the differences in richness between communities [13]. To avoid this disturbance, beta diversity patterns are seen to be influenced by two distinct processes, namely species turnover and nestedness [14,15]. Species turnover represents the replacement of species between different communities, and species nestedness shows that the species composition of one community is a subset of another community [16]. In practice, there may be multiple ecological processes that control species turnover and nestedness patterns, or there may be dominant factors with high explanatory rates among multiple ecological processes. Beta diversity decomposition can clarify the driving forces affecting the community structure from different perspectives, and we can use them to consider more rational biodiversity conservation strategies.

Recently, the sharp decline of biodiversity in the context of global climate change has attracted widespread attention from ecologists [17], and it has become a research hotspot in ecology. Regarding plant diversity, the coexistence mechanism and diversity characteristics of species respond well to environmental processes such as latitude [18,19], altitude [20,21], and drought stress [22]. Surface water disturbance and water stress caused by groundwater depth are the two main factors affecting riparian vegetation patterns [23,24]. Surface water disturbance can also be understood as flood pulse, which refers to the sudden fluctuation of the water volume of a river during a flood resulting in overflow [25]. Flood pulses are the main driver for maintaining the survival and sustaining productivity of river-floodplain systems. Periodic flood pulses can result in the adaptation of organisms and efficient use of floodplain areas [26]. In desert ecosystems in arid and semi-arid regions, surface water resources are severely scarce in most areas. Groundwater enrichment is mainly controlled by stratigraphic lithology and stratigraphic structures, and its dynamics is strongly linked to the evolution of surface processes, which is one of the key drivers that determine the growth status of vegetation, the establishment or loss of populations, and the existence or extinction of oases in desert areas [27,28]. For areas where surface water processes are present, three adaptation mechanisms are thought to occur in plant communities affected by a river overflow. Some studies believe that the vegetation cover increases with the frequency, flow, and intensity of flooding [29]. Moreover, in specific watersheds with high hydrological variability, extreme hydrological events can lead to unpredictable growth patterns of organisms and the coexistence of plants are less related to surface water disturbances [30]. Recently conducted studies related to the intermediate disturbance hypothesis illustrate the specific role of surface water disturbance—a moderate level of disturbance can maintain higher levels of community diversity [31,32].

Current studies on surface water disturbance at different scales have mostly focused on temperate or tropical forests [33–36]. In contrast, in desert ecosystems where short-lived plants do not exist, a perspective that focuses only on comparing vegetation changes before and after diffuse overflow can lead researchers to overlook the cumulative effects of surface water on perennial plants. Simultaneously, most studies on desert riparian forest communities only consider groundwater depth as the dominant factor of environmental heterogeneity, but this is not the only water source affecting desert plant colonization in inland river basins in arid zones. An oasis is a unique geographical landscape in arid and semi-arid regions. The spatial distribution pattern of water resources and the form of recharge determine the oasis scale and the growth of natural vegetation [37]. However, the response mechanisms of the oasis community structure to surface water at the local scale are unclear. In this study, we selected the Daliyabuyi Oasis located in the hinterland of the Taklamakan Desert and used the method of establishing surface water and groundwater gradients at the end of the Kriya River to analyze the driving mechanisms of the formation of beta diversity patterns in the oasis community. By exploring the relative importance of the components of beta diversity after decomposition and their causes, we aim to provide a reference for biodiversity conservation and related research.

## Materials and methods

### Study area

**Fig 1:**
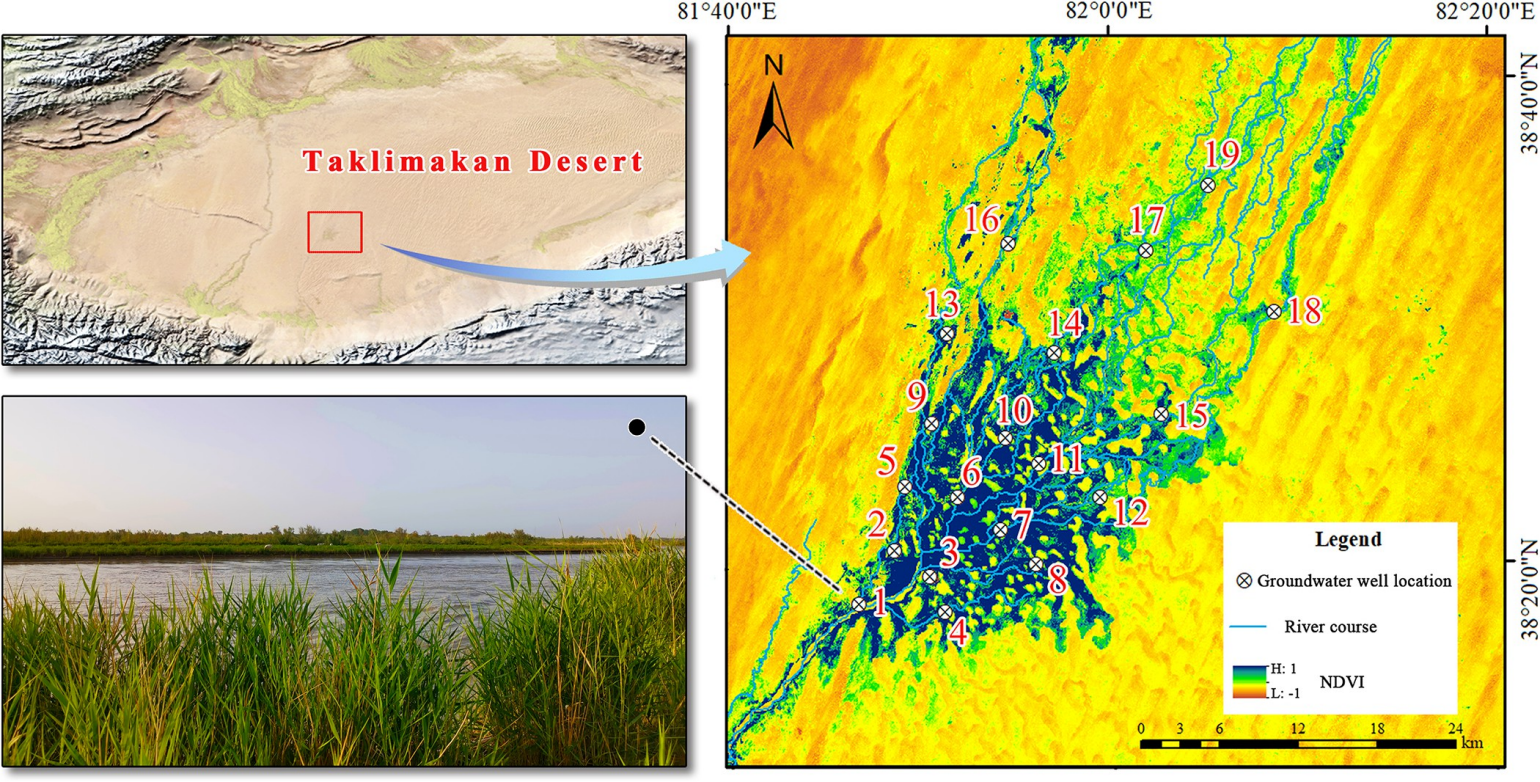
Overview of the study area and sample site selection. The maps are downloaded from Natural Earth (http://www.naturalearthdata.com/) and USGS EROS (http://eros.usgs.gov/). Because the map downloaded from these websites is free and open to scholars, our study does not need to supply a copyright notice.

Daliyabuyi Oasis is located 38°16′–38°37′ N, 81°40′–82°20′ E, in the hinterland of Taklamakan Desert, the second largest flowing desert in the world, and is the terminal oasis of Kriya River, the second largest river at the southern edge of the Tarim Basin in China. The oasis has relatively primitive features due to its isolation and inaccessibility with little human interference. The oasis is between 1100 and 1300 m above sea level, with a total area of about 342 km^2^. The region has a warm temperate arid desert climate, with annual precipitation of less than 20 mm and potential evaporation of more than 2000 mm [38]. With an average annual temperature of 12.1°C, a large temperature difference between day and night, and an extremely arid climate, the natural plant species are relatively poor and consist mainly of perennial species such as *Populus euphratica*, *Tamarix chinensis*, *Alhagi sparsifolia*, and *Phragmites australis*. The interior of the oasis has an intricate anastomosed stream deposit feature [39], and there are periods of high water and withered water annually, different intensity of surface water overflow in some areas during community succession. The project research group built groundwater monitoring wells inside the oasis, providing an ideal site for community ecology studies under surface water and groundwater gradients (Fig 1). The study area is our field experiment station in the Taklamakan Desert hinterland. This is a public area where no endangered or protected species are present, and no permission is required to enter the field site to perform vegetation surveys.

### Site selection and community survey

We set up seven belt transects at the end of 2018, from south to north in the Daliyabuyi oasis, which were nearly perpendicular to the river, and several groundwater monitoring wells were constructed in the belt transect according to the shape and area of the oasis. From June to July 2021, based on the locations of the groundwater monitoring wells that had been constructed near the oasis river channel, we selected areas with a continuous distribution of vegetation to set up nineteen 50 m × 50 m sample plots. The vegetation survey work was conducted within each sample plot, and the species name, number, height, crown width, and other indicators of each species were recorded. For areas where herbaceous plants grew abundantly, twenty small herbaceous quadrats of 1 m × 1 m were randomly laid within the large sample, and the species, height, cover, and abundance of herbaceous plants were investigated and converted into the total number of individuals in the large sample. After the survey, GPS positioning was used to record the location information such as latitude and longitude of each site.

### Soil collection and analysis

Soil samples were collected from six layers (0–5 cm, 5–20 cm, 20–40 cm, 40–60 cm, 60–80 cm, 80–100 cm) from three soil profiles selected at a depth of 1 m in each sample plot. Each layer of soil from the three soil profiles was thoroughly mixed as a representative soil sample of the overall community level. The soil was air-dried in aluminum boxes and plastic bags and returned to the laboratory, ground, and sieved to determine water content, pH, total dissolved solids, organic matter, total nitrogen, and total phosphorus.

### Surface water, groundwater gradient division

In this study, satellite remote sensing technology was used to obtain the water indices of Landsat 8 data in the past five years, and after threshold segmentation, the distribution law and frequency characteristics of surface water during interannual variation were clarified [40]. The purpose of this method was to quantify the disturbance effect of surface water on the community, therefore the formula was established as follows:

$$SWD=ln\sum_{i=1}^{S}w_{i}$$ (1)

**Fig 2:**
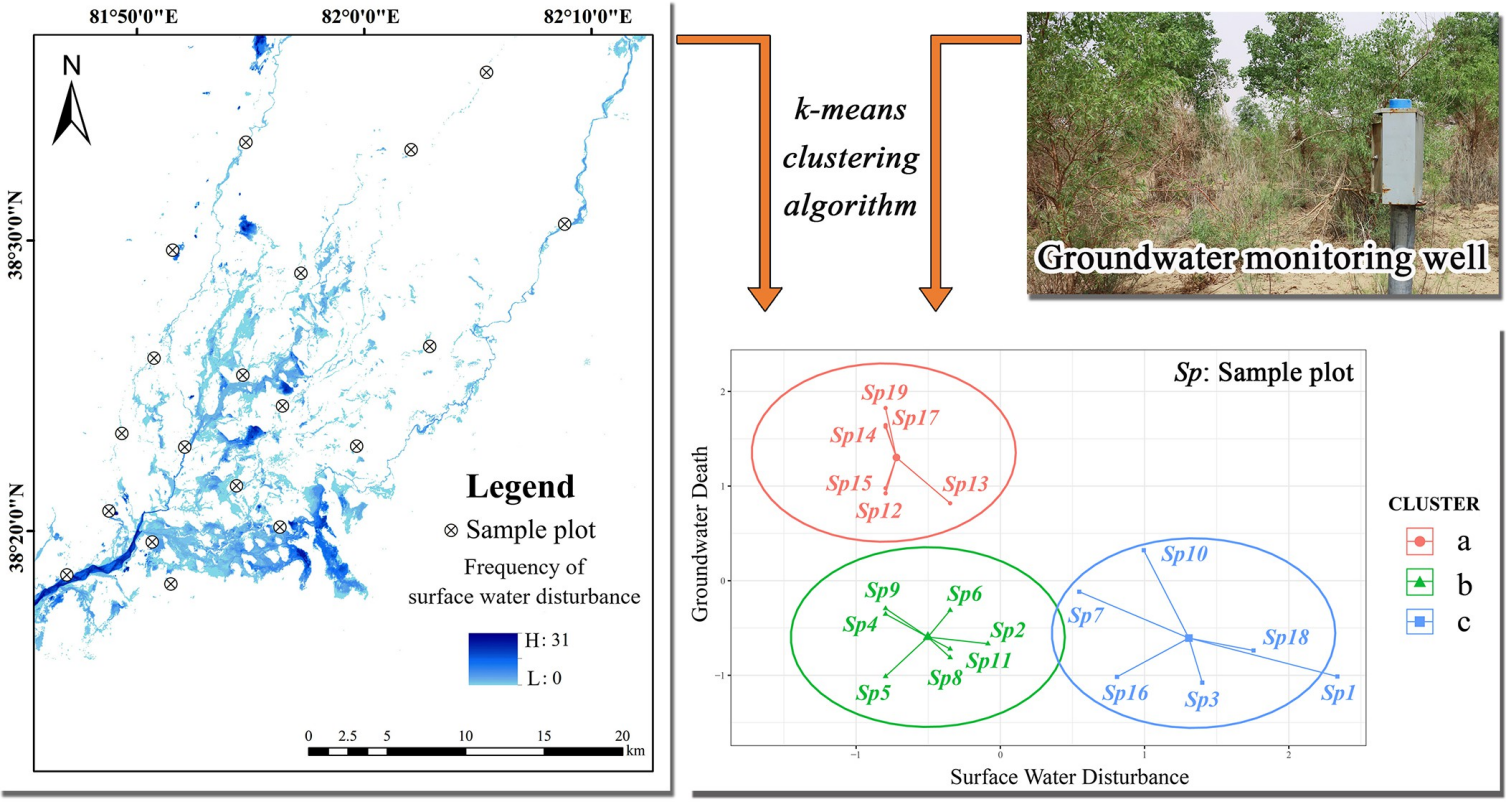
K-mean clustering results of surface water and groundwater. Remote Sensing Image is downloaded from USGS EROS (http://eros.usgs.gov/). Because the Image downloaded from this website is free and open to scholars, our study does not need to supply a copyright notice.

To fully consider the error of handheld GPS in the sample plot positioning process, *SWD* is the degree of surface water disturbance after taking into account the frequency of river overflow and the volume of overflow, *S* represents the number of surface water pixels within 1 ha of the sample site, and *w*_*i*_ represents the frequency characteristics of the *i*th pixels in the monitoring time frame. The higher the *SWD* value, the greater the degree of surface water disturbance. The degree of surface water and groundwater disturbance of the oasis community was divided into gradients using K-means clustering by combing groundwater depth data from the growing season (April to October) when the groundwater monitoring wells were constructed (Fig 2).

### Species beta diversity index

Beta diversity of species was calculated using the multidegree based Sørensen index (βsør) as a beta diversity measure between paired communities, combined with a refinement of this method by Baselga, using the ‘betapart’ package in R to decompose into species turnover (βsim) and species nestedness (βsne) components [41]. The specific calculation formula is as follows:

βsør=(b+c)/(2a+b+c) (2)

βsim=[min(b,c)]/[a+min(b,c)] (3)

βsne=[|b−c|/(2a+b+c)]×{a/[a+min(b,c)]} (4)

In the formula, *a* is the number of common species, *b* and *c* are the number of species unique to each of the two communities.

### Beta diversity driver analysis

Prior to the analysis of beta diversity drivers, DCA analysis of the response variables using the ‘vegan’ package in R4.1.1 showed that each axis length was less than 3; therefore, this study used a redundancy analysis method based on a linear model for the analysis of beta diversity drivers within and among surface water and groundwater gradients. A covariance test is required to eliminate the variance inflation factor (VIF > 10), considering the possible covariance problem between different environmental factors. The screened environmental factors were normalized and imported into CANOCO 5.0 for redundancy analysis. To find the degree of contribution of a single explanatory variable to the response variable, the rdacca.hp function package was used to run hierarchical and variance partitioning [42] to assess the importance of individual explanatory variables and co-explanatory variables in the formation of beta diversity patterns.

For the possible attenuation relationship between the similarity of communities and spatial distance, the role of spatial distance in the oasis community formation can be elucidated through the quantitative analysis of distance decay curves [43]. In this study, a linear fit was performed using the log-transformed community similarity (1-βsør) to the spatial distance, and the resulting slope is the eigenvalue of the distance decay.

## Results

### Characteristics of intra-gradient beta diversity and its components

Species beta diversity tends to increase under the three gradient sequences (a, b, c) formed by surface water and groundwater. The species turnover component (72.66%) mainly contributed to the beta diversity among communities within the third gradient (c). Beta diversity among communities within the first gradient (a) is contributed by species turnover and species nestedness components, accounting for 52.94% and 47.06%, respectively, with the turnover component slightly higher than the nestedness component. Beta diversity among communities within the second gradient (b) was similarly contributed by species turnover and nestedness components. However, in contrast to the first gradient (a), the contribution of the nested component within this gradient (53.31%) is slightly higher than that of the turnover component (46.69%). Nevertheless, the first gradient (a) and second gradient (b) species turnover and nestedness of the two components are not substantially different (Table 1).

**Table 1:** Species beta diversity and its component characteristics within surface water and groundwater gradients (mean±SE).

Gradient	Beta diversity (βsør)	Species turnover (βsim)	Species nestedness (βsne)
a	0.283±0.072	0.150±0.059	0.133±0.037
b	0.408±0.052	0.191±0.057	0.218±0.038
c	0.505±0.046	0.367±0.070	0.138±0.032

a: Low surface water disturbance and Deep groundwater depth; b: Low surface water disturbance and Shallow groundwater depth; c: High surface water disturbance and Shallow groundwater depth.

### Characteristics of inter-gradient beta diversity and its components

Regarding the beta diversity of community species among water resource gradients, beta diversity was highest between the second and third gradients (b-c), followed by the first and third gradients (a-c), both of which were nearly equal. The beta diversity between the first and second gradients (a-b) is smaller than the first two. According to the variation characteristics of the two components of species turnover and nestedness, it can be deduced that when the degree of surface water disturbance is low, and there is a gradient difference from deep to shallow groundwater depth, the highest percentage of the component of species nestedness is 54.42%. When both groundwater depths are shallow, and there is a gradient difference in surface water disturbance from low to high, the component of species turnover is higher than the nestedness component, 59.48% vs. 40.52%, respectively. When the degree of surface water disturbance changed from low to high, the depth of groundwater burial changed from deep to shallow, slightly increasing the gap between the two components of species turnover and nestedness, accounting for 60.23% and 39.77%, respectively (Table 2).

**Table 2:** Species beta diversity and its component characteristics among surface water and groundwater gradients (mean±SE).

Gradient	Beta diversity (βsør)	Species turnover (βsim)	Species nestedness (βsne)
a-b	0.327±0.040	0.149±0.035	0.178±0.027
b-c	0.412±0.033	0.245±0.039	0.167±0.022
a-c	0.411±0.036	0.248±0.042	0.164±0.024

a: Low surface water disturbance and deep groundwater depth; b: Low surface water disturbance and shallow groundwater depth; c: High surface water disturbance and shallow groundwater depth.

### Distance decay mechanism of oasis community similarity

**Fig 3:**
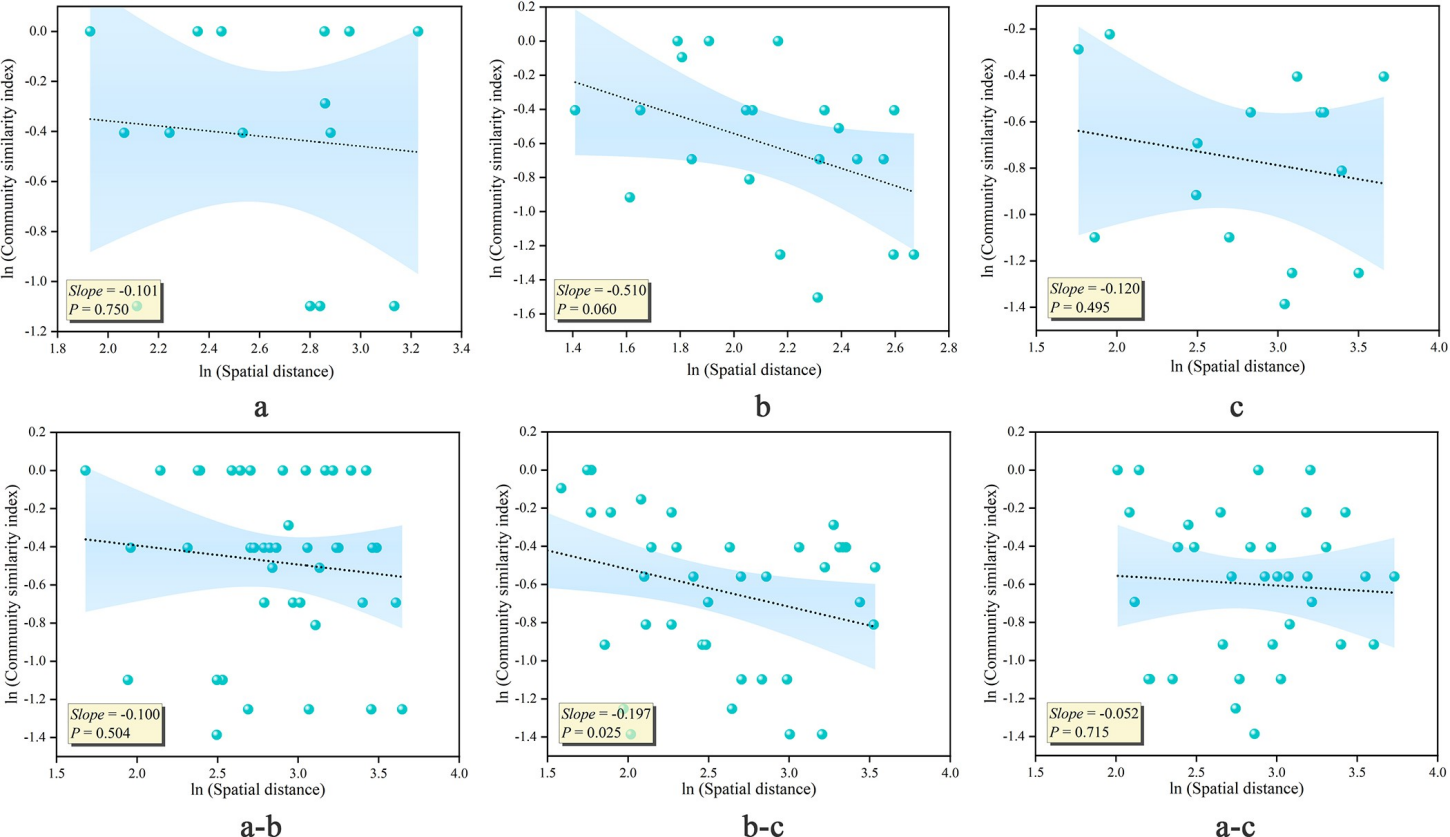
Results of linear fitting of distance decay characteristics of community similarity within and among gradients. (a: Low surface water disturbance and deep groundwater depth; b: Low surface water disturbance and shallow groundwater depth; c: High surface water disturbance and shallow groundwater depth).

According to the linear relationship between the community similarity index (1-βsør) and spatial distance, the community similarity within and among surface water and groundwater gradients decreases with increasing spatial distance (Slope < 0), consistent with the distance decay mechanism of community similarity. However, most linear fits were insignificant (P > 0.05), indicating that although community assembly in the Daliyabuyi oasis was influenced to some extent by diffusion limitation, its role in the gradient experiment was small, and there was no universal law (Fig 3).

### Drivers of intra-gradient species beta diversity patterns

**Fig 4:**
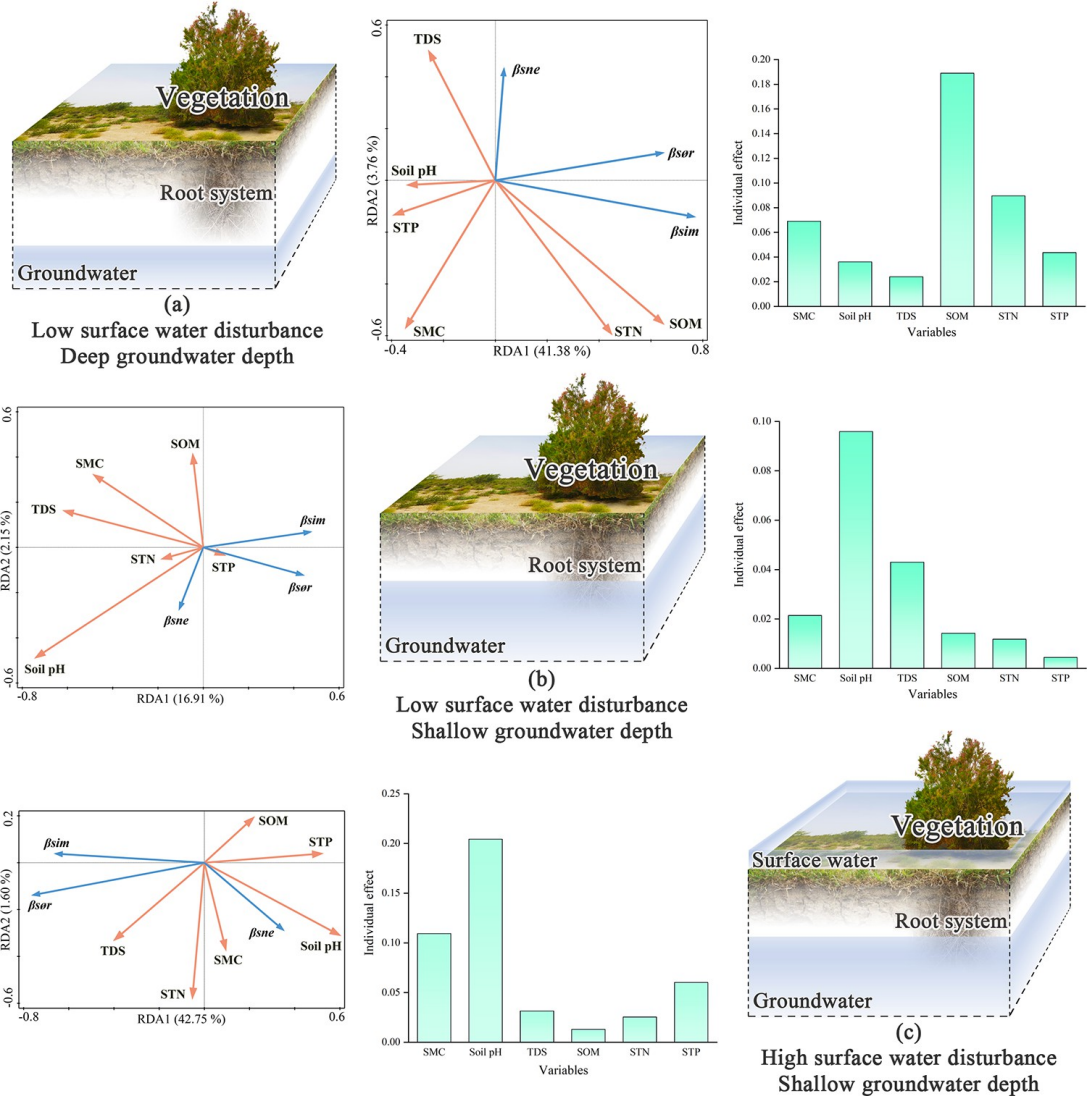
Results of RDA analysis within different surface water and groundwater gradients. (SOM: Soil organic matter; STP: Soil total phosphorus; STN: Soil total nitrogen; SMC; soil water content; TDS: Total dissolved solids).

Within the gradient of low surface water disturbance and deep groundwater depth (a), environmental factors explained 41.38% and 3.76% of the beta diversity pattern characteristics in the first two axes of the RDA analysis, respectively. The individual explanation rate of soil organic matter difference was the highest at 18.90%, and it was positively correlated with the overall beta diversity and species turnover component of the gradient and negatively correlated with the species nestedness component. In the low surface water disturbance and shallow groundwater depth gradient (b), environmental factor differences explained 16.91% and 2.15% of the beta diversity pattern characteristics in the first and second axes, respectively. The soil pH difference in this gradient had the highest individual explanation rate, 9.59%, and was negatively correlated with beta diversity and species turnover components, but positively correlated with species nestedness components; Within the high surface water disturbance and shallow groundwater depth gradients (c), soil environmental factors explained 42.75% and 1.60% of the beta diversity pattern characteristics in the first and second axes, respectively. Within this gradient, the individual explanation rate of soil pH difference was still the highest at 20.44%. Soil pH differences were negatively correlated with beta diversity and species turnover component and positively correlated with species nestedness component (Fig 4).

### Drivers of inter-gradient species beta diversity patterns

The biotopes were not significantly disturbed by surface water between the first and second gradients (a-b). The groundwater depth differences under this condition significantly impacted the formation of species beta diversity patterns, with an individual explanation rate of 8.37%. It was negatively correlated with beta diversity and species turnover components, positively correlated with species nestedness components, and was the main driving force regarding changes in beta diversity patterns between the first and second gradients.

Between the second and third gradients (b-c), the biotopes were in a state of consistent shallow groundwater depths, but differences in surface water disturbance were not the main driving force behind changes in the beta diversity pattern. The soil pH difference between the two gradients had the highest individual explanation rate, 5.13%, and was negatively correlated with the overall beta diversity and species turnover component and positively correlated with the species nestedness component.

**Fig 5:**
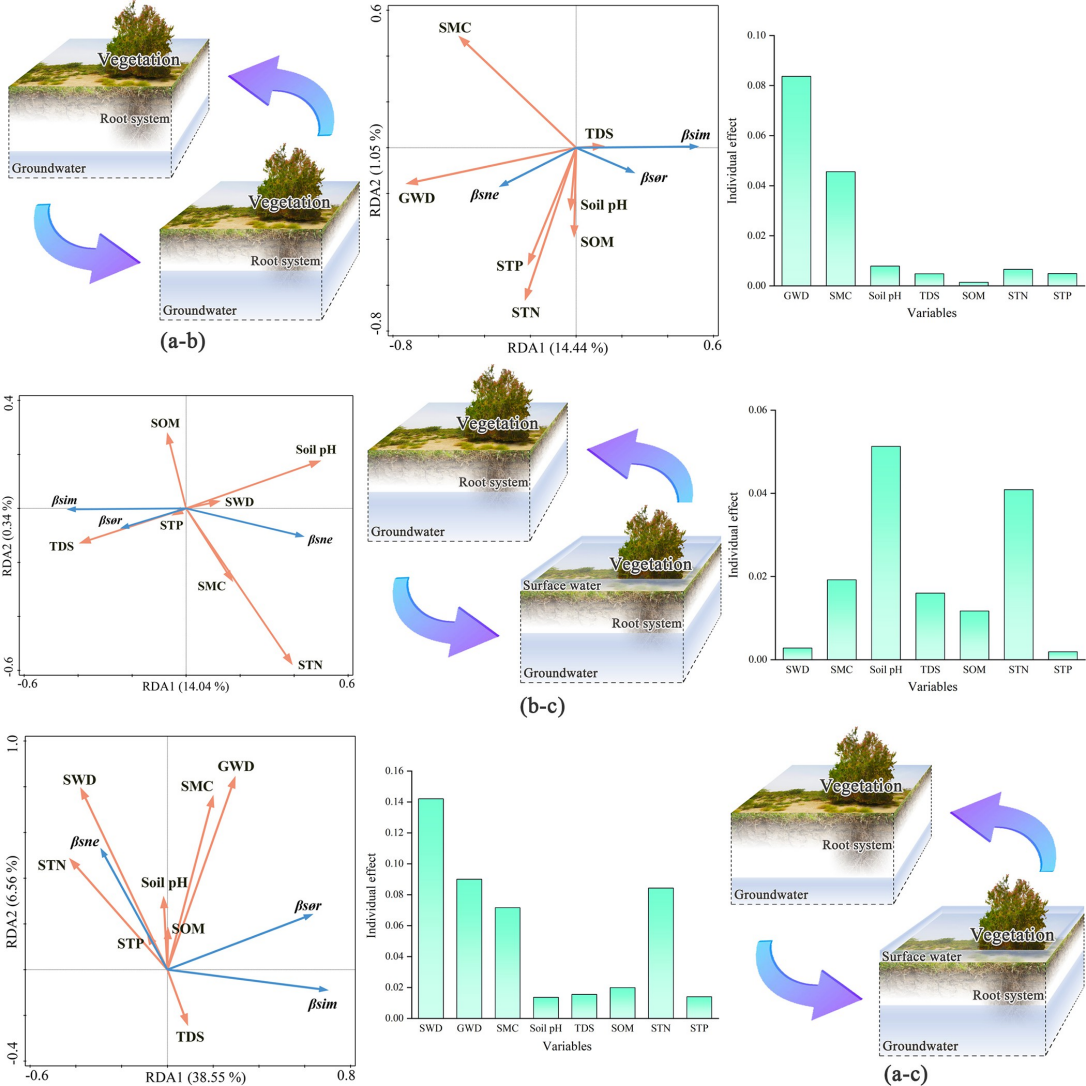
Results of RDA analysis among different surface water and groundwater gradients. (a: Low surface water disturbance and deep groundwater depth; b: Low surface water disturbance and shallow groundwater depth; c: High surface water disturbance and shallow groundwater depth; SWD: Surface water disturbance; GWD: Groundwater depth; SOM: Soil organic matter; STP: Soil total phosphorus; STN: Soil total nitrogen; SMC: Soil water content; TDS: Total dissolved solids).

Surface water disturbance and groundwater depth were different between the first and third gradients, and they were the main driving forces for the formation of the beta diversity pattern between the two gradients. The surface water disturbance difference has an individual explanation rate of 14.21%, which is positively correlated with the species nestedness component in the RDA analysis, negatively correlated with the species turnover component, and has a low correlation with the beta diversity. Groundwater depth differences had a 9.00% individual explanation rate and were positively correlated with overall beta diversity and species nestedness components in RDA analysis but were less correlated with species turnover components (Fig 5).

### Response of beta diversity and its components to environmental variables

**Fig 6:**
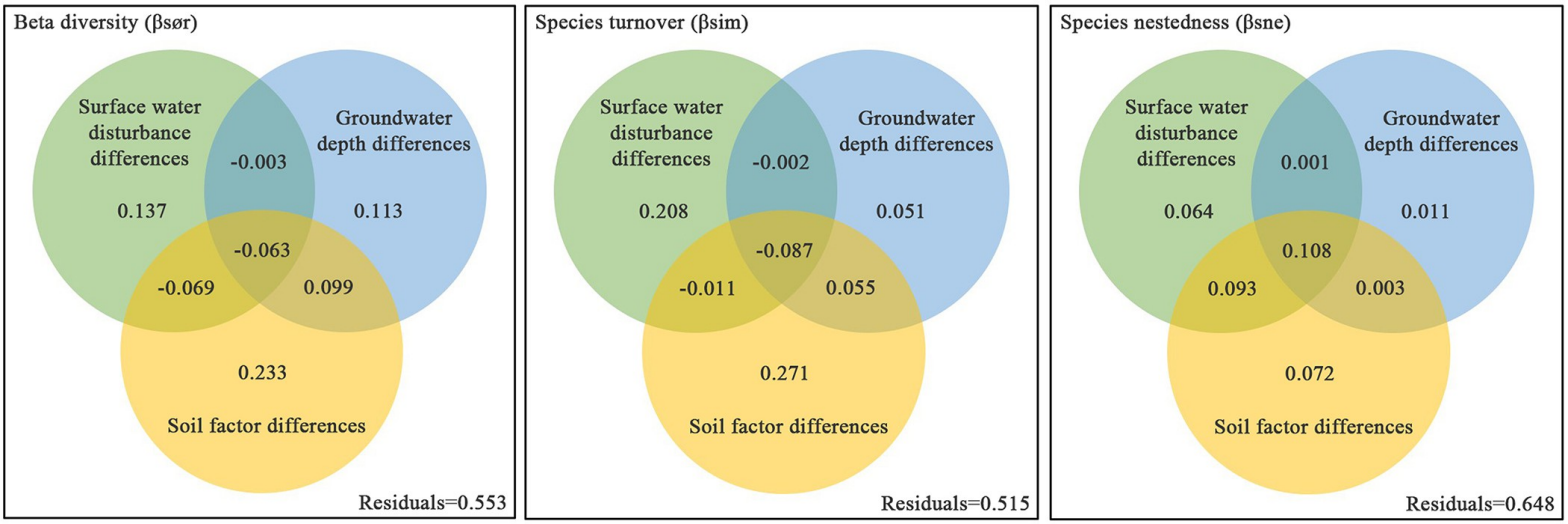
Variance partitioning of beta diversity and its components in the presence of simultaneous surface water disturbance and groundwater depth differences.

The individual explanations for environmental variables mentioned above are the individual effects of each explanatory variable after the hierarchical partitioning. To better understand the unique and common effects among the explanatory variables, we selected gradients a-c with surface water disturbance and groundwater depth difference. We used the variance partitioning method to determine the response of beta diversity and its components to different explanatory variables (Fig 6).

In the variance partitioning, the unique explanation proportion of soil factor differences is slightly higher because it considers various soil physicochemical properties under the environmental gradient. Therefore, the unique explanatory power of soil factor differences in the variance partitioning process is a collection of various soil physical and chemical properties. The influence of surface water disturbance differences on the species beta diversity pattern characteristics is slightly higher than that of groundwater depth differences (0.137 > 0.113), and there is a 9.9% common explanation part between groundwater depth and soil factor differences. Regarding species turnover components, the part of surface water disturbance differences that can be explained as unique is much higher than that of groundwater depth differences (0.208 > 0.051), and there is a 5.5% common explanation part between groundwater depth and soil factor differences. For species nestedness components, the part of surface water disturbance differences that can be explained as unique is higher than groundwater depth differences (0.064 > 0.011). Furthermore, there is a 0.1% common explanation part between surface water and groundwater, a 9.3% common explanation part between surface water and soil factors, and a 0.3% common explanation part between groundwater and soil factors, making a total of 10.8% common explanation part among the three.

Because of the above results, we set an explanation rate of 0.100 as the benchmark value, and environmental variables greater than the benchmark value were the dominant factors driving beta diversity and its components. The results show that when multiple soil factors are aggregated as a whole soil environmental variable, beta diversity is independently influenced by surface water disturbance, groundwater depth, and soil environmental differences. Species turnover components are mainly independently influenced by surface water disturbance and soil environmental differences; The species nestedness components are mainly common influenced by the surface water disturbance, groundwater depth, and soil environment differences.

## Discussion

### Dependence of intra-gradient beta diversity on surface water and groundwater

The beta diversity analysis of species is divided into two categories: intra-gradient and inter-gradient for discussion. The directionality of beta diversity within a gradient is not always clear. The variation in community structure within a given gradient tends to reflect more closely the dispersion of species distribution characteristics and can effectively determine the true impact of environmental variables on habitat change without considering community type [44,45]. Owing to the effect of this condition, the explanation rate of environmental factors within the same gradient range cannot directly imply a positive or negative effect on the community, but rather refers to the importance of environmental variables in causing differences in species composition between communities. The study showed that as groundwater depth became shallower, the dominant factor driving the beta diversity pattern shifted from soil organic matter to pH. On this basis, with the intensification of surface water disturbance, the individual explanation rate of pH also increases, a result that is consistent with the results of a study reported by Glaser et al. in northern Minnesota rivers [46]. This is because flooding and drought processes can directly affect physicochemical properties of the soil, especially soil pH, which further affects plant communities [47]. In addition, it has also been pointed out that the effect of soil heterogeneity on plant species diversity depends on changes in soil nutrients and pH, and the opposite effects of these two on species diversity are commonly found in wild plant communities [48]. In desert ecosystems, changes in pH are more likely to negatively affect the community, thereby reducing its stability [49]. In conclusion, the soil pH changes play a substantial role in desert-wetland ecosystem communities, and this provides some help for deepening the understanding of the maintenance mechanism of diversity under arid and semi-arid conditions.

### Dependence of inter-gradient beta diversity on surface water and groundwater

**Fig 7:**
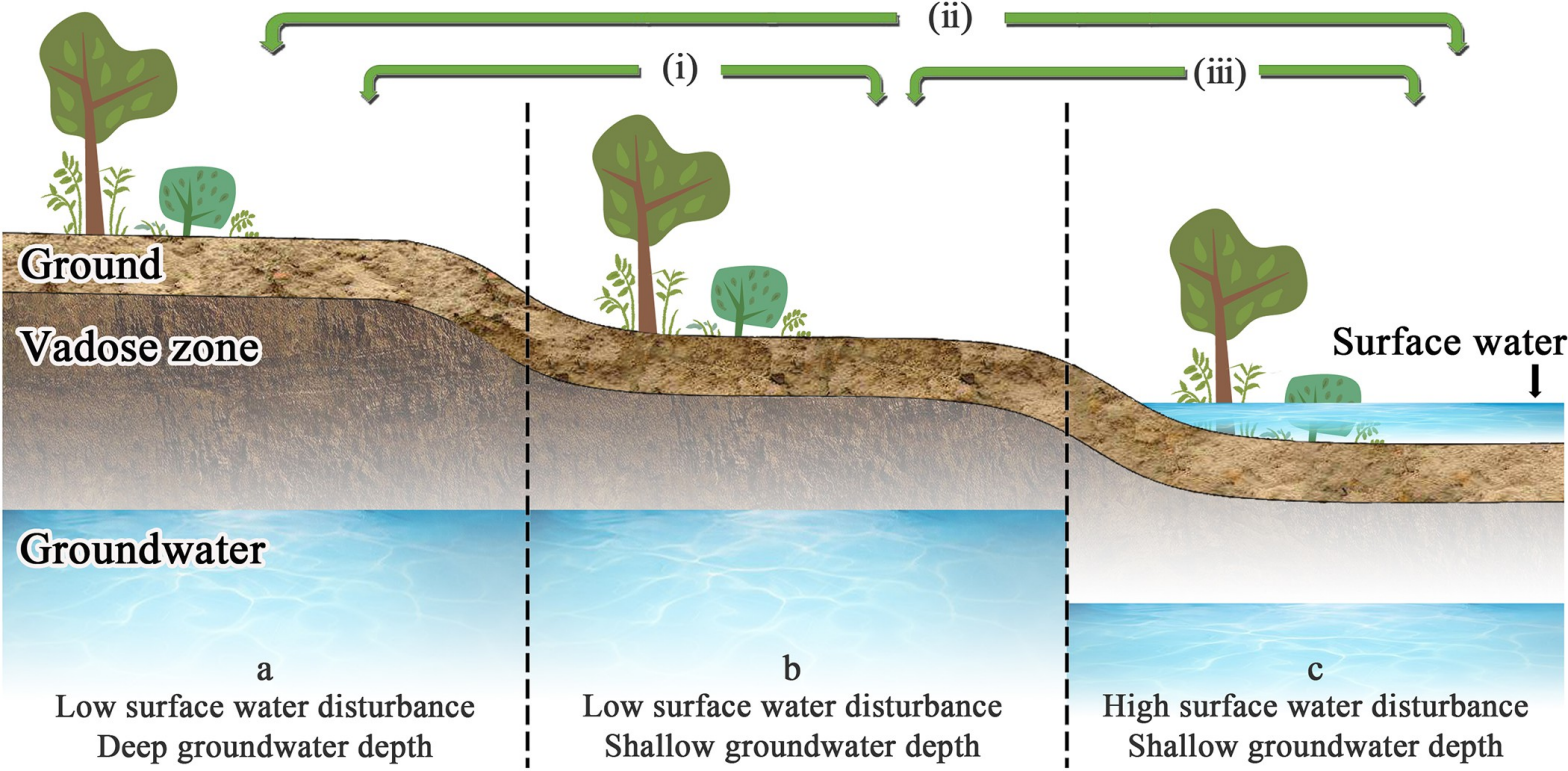
Conceptual diagram of the dependence of beta diversity among gradients on surface water and groundwater. (i, ⅱ, and ⅲ represent the three driving mechanisms of the oasis community under the influence of surface water and groundwater, respectively).

Compared with species beta diversity within gradients, this part of the study has focused on community structure changes along surface water and groundwater gradients. Based on the results from this study, three mechanisms for the formation of oasis community differences in the desert hinterland were revealed. (i) When the oasis biotope is not disturbed by surface water, groundwater depth is the key factor driving the transition of community structure. (ⅱ) When a biotope with a harsh initial environment and no water resources directly transitions to a biotope with abundant surface water and groundwater resources, the difference in surface water disturbance plays an important role in the process of community assembly. (ⅲ) However, when the uplift of groundwater depth has impacted the habitat, the growth state of species in the community is more suitable for the environmental conditions of shallow groundwater depth. The impact of surface water disturbance on the community is negligible (Fig 7).

In this study, when environmental variables were used to reveal the driving mechanism of the first and second gradients (a-b), the second and third gradients (b-c) on the beta diversity and its composition pattern, the overall RDA analysis showed that the interpretation rate was low. Unconsidered ecological processes between these two gradients, such as regional species pool [50], ecological drift [51] and many specific selection processes [52], may also be potential drivers of diversity patterns. The purpose of this study was to rely on the difference in the influence of surface water disturbance and groundwater depth, focusing on the relative importance of existing environmental factors under different gradients and the beta diversity pattern driven by environmental variables. The relative contribution of environmental factors to community assembly is not affected by other ecological processes, while the unknown ecological processes and their quantification methods need to be further studied.

### Species turnover and nestedness between surface water and groundwater gradients

Potential mechanisms leading to species turnover are generally considered to include environmental filtering, competition exclusion, and geographic isolation [53–55]. In contrast, species nestedness usually occurs in communities with nested habitat conditions subjected to selective extinction and migration processes [56]. Competition refers to the relationship between two or more species that hinder and restrict each other so that the relative fitness between coexisting species tends to be equal, which in turn affects the community assembly process [57,58]. According to studies on phylogenetic ecology, the phylogenetic structure shows a dispersion pattern (S1 File). The results also show the that the effect of competitive exclusion under these research conditions is greater than that of environmental filtering, and therefore this process is the main driving force affecting the pattern of species turnover. Influenced by the scale of the oasis in the study area, the distribution of dry river channels, and the low dispersibility of plants, the distance attenuation mechanism of community similarity does not have a significant general law, and the two mechanisms of geographic isolation and selective migration are not applicable in this oasis. According to the variance partitioning results, the dominant factor affecting species nestedness is not the unique effect of some environmental variables but the common effect of surface water, groundwater, and soil environment. Simultaneously, there are also common effects between pairs of environmental variables. The explanatory power of multiple environmental differences shapes the diversity of environmental pressures in natural habitats, which then reinforces the possibility that species nestedness is explained by selective extinction.

## Conclusions

In the desert hinterland oasis formed at the end of an inland river in an arid area, the differences in surface water disturbance and groundwater depth can create three different gradients. The environmental factor dominating beta diversity changed from soil organic matter to soil pH as groundwater depth became shallower within different gradients. The intensification of surface water disturbance further increases the effect of soil pH, which becomes a key explanatory factor for distinguishing the abundance of water resources in oasis communities.

In addition, we also focused on the variation characteristics of community structure along the water gradients and considered the effects of oasis surface water disturbance and groundwater depth differences on community assembly. This study shows that differences in groundwater depth can dominate the shift in community structure when surface water disturbances are all low. Surface water disturbance mainly affects communities in deep groundwater depth areas where the original water resources are extremely scarce. However, when the groundwater depth of the habitats was shallow, the community adapted to the shallow groundwater depth habitat, and the difference in surface water disturbance had insubstantial effect on community structure changes. For the two components of beta diversity, the main drivers of species turnover pattern was the unique effects of surface water disturbance and soil environmental differences, and the main driver of species nestedness was the common effect of multiple environmental pressures. Notably, the communities dwelling in harsh water resource environments are not entirely nested subsets of the communities with more abundant water resources. Therefore, when controlling the degradation of desert oases and delineating reasonable priority protection areas, areas subjected to severe drought due to water stress should also be considered.

This study confirms that surface water and groundwater play different roles in constructing desert oasis communities in inland river basins in arid regions. A comprehensive study of the difference in the impact of the two can clarify the formation mechanism of the oasis community. Equal focus on both factors can promote the protection of desert ecosystems in a multi-faceted way and prevent further biodiversity loss. The results of this study indicate that increasing the disturbance of surface water in dry areas by means of river flooding will aid in promoting vegetation restoration and alleviating the degradation of oases. This view also supports the ecological water transfer policy adopted for China’s Tarim River since the 21st century. Simultaneously, the decomposition of beta diversity provides a new perspective for community assembly driven by surface water and groundwater and has profound significance in quantitatively evaluating the impact of the environment on changes in the community structure.

## Supporting information

S1 Fig The regression relationship between surface water disturbance and groundwater depth. (JPG)

S1 Table Coordinates of the 19 sample plots. (PDF)

S1 File Phylogenetic characteristics of oasis communities in the desert hinterland. (PDF)
