# Supplementary figures and images for: The combined effect of surface water and groundwater on environmental heterogeneity reveals the basis of beta diversity pattern in desert oasis communities
Source: PLoS One. 2022 Dec 27;17(12):e0279704. doi: 10.1371/journal.pone.0279704 (PMC9794059; doi:10.1371/journal.pone.0279704)

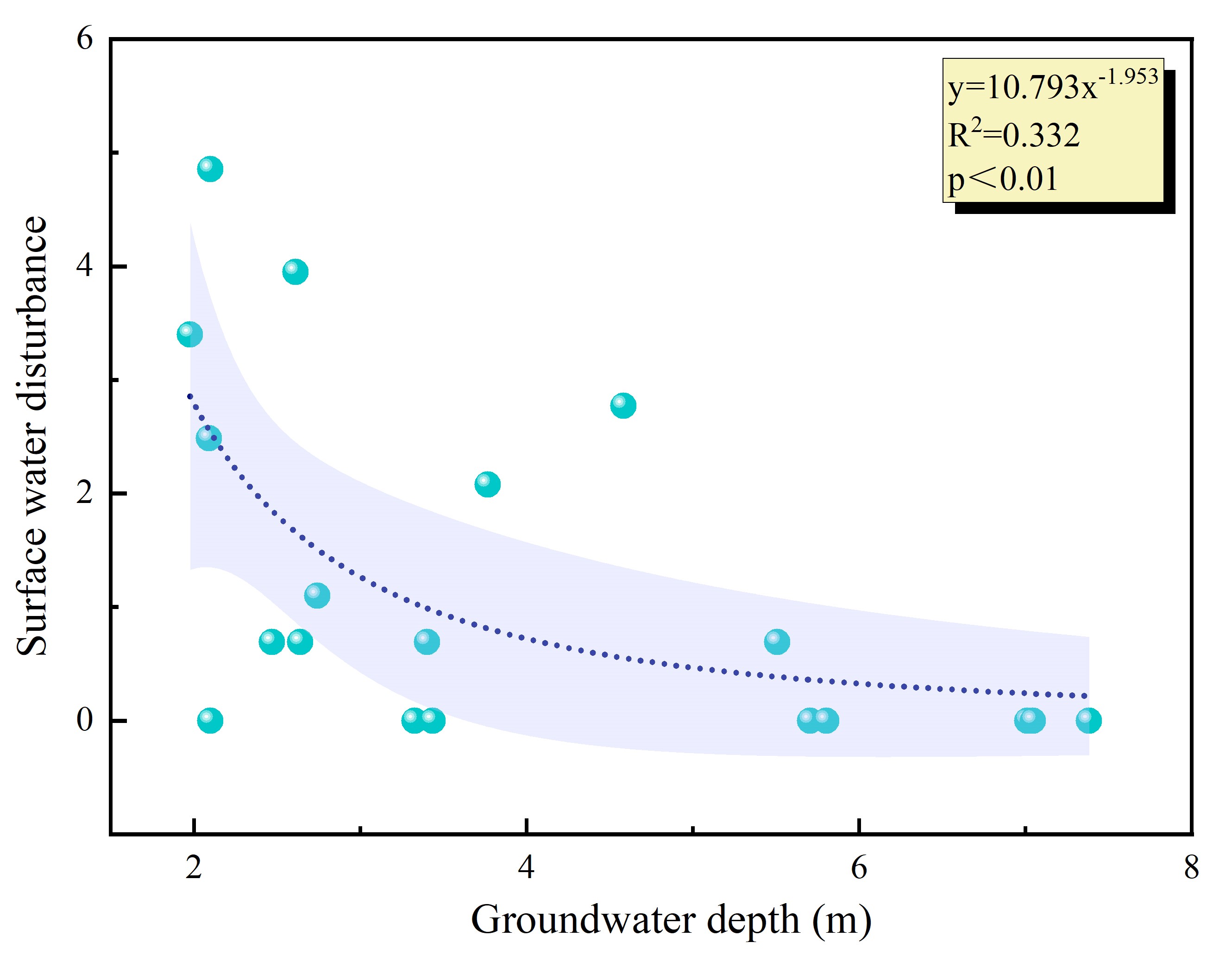

Supplement: S1 Fig — (JPG) [file pone.0279704.s001.jpg]
