# Supplementary material for: The combined effect of surface water and groundwater on environmental heterogeneity reveals the basis of beta diversity pattern in desert oasis communities
Source: PLoS One. 2022 Dec 27;17(12):e0279704. doi: 10.1371/journal.pone.0279704 (PMC9794059; doi:10.1371/journal.pone.0279704)
Supplement: S1 Table — (PDF) [file pone.0279704.s002.pdf]

S3 Table. Coordinates of the 19 sample plots

Sample plot number	Longitude	Latitude
1	81°46′36.982″	38°18′28.690″
2	81°48′29.285″	38°20′39.850″
3	81°50′21.871″	38°19′35.19″
4	81°51′9.518″	38°18′8.026″
5	81°49′04.85″	38°23′19.821″
6	81°51′49.615″	38°22′50.633″
7	81°54′4.493″	38°21′29.577″
8	81°55′57.270″	38°20′3.392″
9	81°50′31.014″	38°25′54.413″
10	81°54′23.658″	38°25′18.111″
11	81°56′4.36″	38°24′21.996″
12	81°59′20.854″	38°22′49.152″
13	81°51′22.36″	38°29′38.24″
14	81°56′59.362″	38°28′47.137″
15	82°02′35.345″	38°26′13.277″
16	81°54′37.82″	38°33′18.52″
17	82°01′52.127″	38°32′59.851″
18	82°08′33.557″	38°30′21.667″
19	82° 05′13.131″	38°35′37.73″
