# Supplementary material for: The combined effect of surface water and groundwater on environmental heterogeneity reveals the basis of beta diversity pattern in desert oasis communities
Source: PLoS One. 2022 Dec 27;17(12):e0279704. doi: 10.1371/journal.pone.0279704 (PMC9794059; doi:10.1371/journal.pone.0279704)
Supplement: S1 File — (PDF) [file pone.0279704.s003.pdf]

Supporting information

S1 File. Phylogenetic characteristics of oasis communities in the desert hinterland.

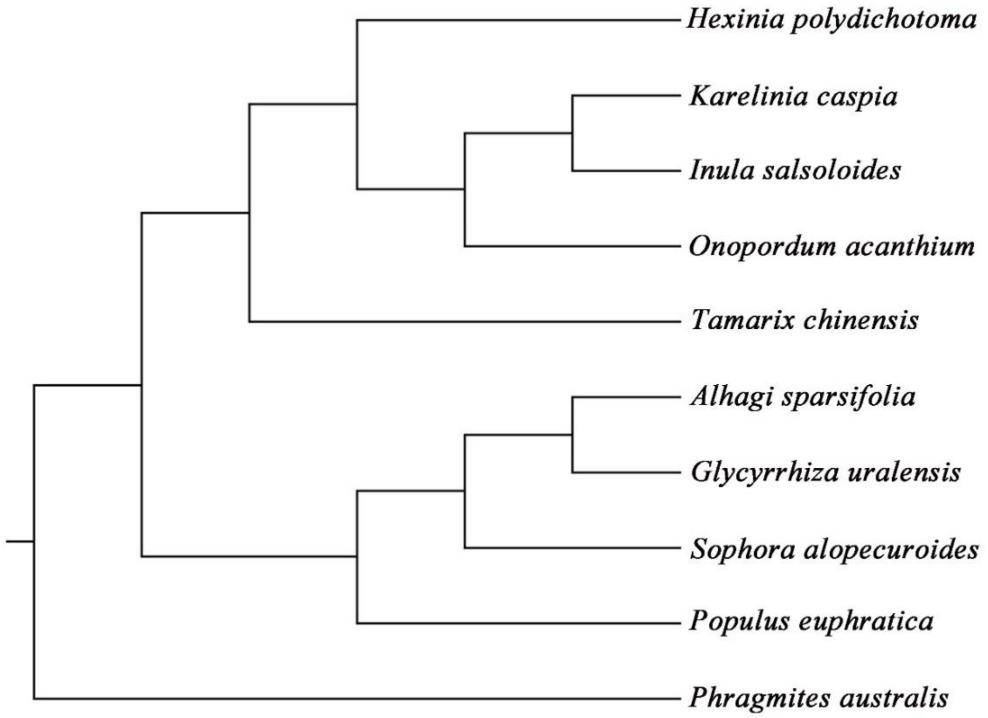

S1 Fig. A phylogenetic tree of oasis species in the desert hinterland

S1 Table. Phylogenetic diversity of oasis communities (mean±SE)

Gradient	Phylogenetic diversity index (PD)	Net relatedness index (NRI)	Net nearest taxa index (NTI)
a	326.29±43.22	-0.43±0.10	-0.51±0.18
b	427.02±63.11	-0.74±0.12	-0.89±0.19
c	418.34±41.12	-0.68±0.10	-0.75±0.18

The phylogenetic structures under different gradients did not show aggregation characteristics, and the role of competitive exclusion in community assembly was greater than that of environmental filtering.
